# Phytohormones regulate the non-redundant response of ω-3 fatty acid desaturases to low temperatures in *Chorispora bungeana*

**DOI:** 10.1038/s41598-023-29910-4

**Published:** 2023-02-16

**Authors:** Yulan Shi, Sizhong Yang, Zhixing Zhao, Lizhe An

**Affiliations:** 1 Extreme Stress Resistance and Biotechnology Laboratory, Northwest Institute of Eco-Environment and Resources, Chinese Academy of Sciences, Lanzhou, 730000 China; 2 State Key Laboratory of Frozen Soils Engineering, Northwest Institute of Eco-Environment and Resources, Chinese Academy of Sciences, Lanzhou, 730000 China; 3 School of Life Sciences, Lanzhou University, Lanzhou, 730000 China; 4 School of Forestry, Beijing Forestry University, Beijing, 100083 China

**Keywords:** Molecular biology, Plant sciences

## Abstract

To explore the contributions of ω-3 fatty acid desaturases (FADs) to cold stress response in a special cryophyte, *Chorispora bungeana*, two plastidial ω-3 desaturase genes (*CbFAD7*, *CbFAD8*) were cloned and verified in an *Arabidopsis fad7fad8* mutant, before being compared with the microsomal ω-3 desaturase gene (*CbFAD3*). Though these genes were expressed in all tested tissues of *C. bungeana*, *CbFAD7* and *CbFAD8* have the highest expression in leaves, while *CbFAD3 *was mostly expressed in suspension-cultured cells. Low temperatures resulted in significant increases in trienoic fatty acids (TAs), corresponding to the cooperation of *CbFAD3* and *CbFAD8* in cultured cells, and the coordination of *CbFAD7* and *CbFAD8* in leaves. Furthermore, the cold induction of *CbFAD8* in the two systems were increased with decreasing temperature and independently contributed to TAs accumulation at subfreezing temperature. A series of experiments revealed that jasmonie acid and brassinosteroids participated in the cold-responsive expression of ω-3 *CbFAD* genes in both *C. bungeana* cells and leaves, while the phytohormone regulation in leaves was complex with the participation of abscisic acid and gibberellin. These results point to the hormone-regulated non-redundant contributions of ω-3 CbFADs to maintain appropriate level of TAs under low temperatures, which help *C. bungeana* survive in cold environments*.*

## Introduction

Low temperature is one of the major environmental stresses influencing the distribution of plant species. To withstand this stress, plants have developed adaptive mechanisms, which are rather complex and include the regulation of cell components as well as metabolic changes^1,2^. Cell membranes, serving as the boundary and active interface between cells/organelles and their environment, are the major targets of low temperature acclimation^1–3^. Although the structural and functional integrity of cell membranes are usually affected by low temperatures, the membrane integrity can be maintained by fatty acid modification^3,4^. In fact, the content of TAs, represented mainly by C18:3, are improved to a certain extent in response to low temperatures, thus maintaining membrane fluidity and status^5–7^.

The synthesis of TAs is performed by ω-3 FADs through introducing a double bond into the ω-3 position of dienoic fatty acids^1^. It is known that ω-3 FADs are one kind of acyl-lipid desaturases, which could be classified into two types according to cellular localization: The plastid-type desaturase (FAD7 and FAD8) is localized in plastid membranes^8,9^, and the microsome-type desaturase (FAD3) is localized in the endoplasmic reticulum^10^. As one of the important factors for cold response^11^, the expression of ω-3 *FAD* genes have been widely researched in plants. The first finding from maize leaf showed an increase in *ZmFAD8* mRNA accompanied by a decrease in *ZmFAD7* mRNA under 5 °C exposure^12^. Later, relevant studies have been carried out in various plant species, such as birch^13^, *Descurainia sophia*^14^, purslanen^15^, soybean^16^, *Arabidopsis*^17,18^, safflower^19^, *Gossypium raimondii*^20^, *Medicago truncatula*^21^ and rice^22^. However, most of the studies focused on the common plants or crops undergoing chilling temperatures (2–16 °C), little attention has been paid to the cryophytes (typical cold-tolerant plants) surviving the extreme cold conditions. Therefore, it is unclear whether there are differences in the cold response of ω-3 FADs between cryophytes and the other plant species.

Besides that, though some phytohormones, for example abscisic acid (ABA), salicylic acid (SA), jasmonie acid (JA), brassinosteroids (BRs) and gibberellin (GA) were thought to be the signal molecules involved in plant cold response^2,23–26^, we still poorly understand whether these hormones participate in the cold response of ω-3 *FAD* genes. There was only one direct evidence confirmed that JA partially participated in the chilling-induced expression of ω-3 *FAD* genes in *Arabidopsis*^17^. Recently, a study from *Arabidopsis* leaf indicated that AtFAD7 protein levels decreased upon ABA treatment, while AtFAD8 protein levels increased upon cold or JA exposure^27^. Unfortunately, the new findings have not directly proved the influence of ABA and JA on the cold response of AtFAD7 and AtFAD8. So far, the phytohormones that transmit low temperature signals to ω-3 *FAD* genes and regulate their expression have not been identified. Considering that the critical role of hormonal and stress factors in polyunsaturated fatty acid metabolism have been clearly confirmed in rodents and humans^28^, similar studies on plants should attract enough attention. Perhaps the research on cryophytes will help us get more information.

*Chorispora bungeana* (*C. bungeana*) is a perennial cruciferous cryophyte, having a close genetic relationship with *Arabidopsis*^29^. It inhabits periglacial areas (about 3800–3900 m), where experience the bitter cold in winter and the freeze–thaw cycles in summer. To survive in the extreme environment, *C. bungeana* has adapted certain physiological and molecular mechanisms^3,29–35^ instead of special morphological characteristics^36^. Using cell suspension cultures, we found that the cold tolerance of *C. bungeana* was closely related to the accumulation of C18:3, however, the contribution of each ω-3 CbFAD on this progress is unknown^3^.

As a versatile experimental system, plant cell suspension cultures provide a possibility to analyze complex plant physiological processes in a more simplified system compared to the organism *in toto*^37,38^. Therefore, *C. bungeana* suspension-cultured cells are often used to study the physiological and molecular mechanisms of cold tolerance in our lab^3,39–41^. Meanwhile, the regenerated plants of *C. bungeana* are another useful experimental system can meet the research needs on tissue or organism level, in view of the low yield of wild *C. bungeana* and the sterility of cultivated *C. bungeana*^29–31,35^. Given that *CbFAD3* (microsomal) and *CbFAD7*/*CbFAD8* (plastidial) were mostly expressed in suspension-cultured cells and the leaves of regenerated plants, respectively, the experiments of this work were performed on the two materials to extend the analysis from cellular level to tissue level. The common phenomenon from the different analysis systems can be identified as the core adaptive mechanism in *C. bungeana*.

## Results

### cDNA isolation and sequence analysis of *CbFAD7* and *CbFAD8* from *C. bungeana*

After clone and verification, two full length cDNA of 1805 and 1563 bp were obtained and designated as *CbFAD7* (KY069282) and *CbFAD8* (KY069283), respectively. *CbFAD7* contains an ORF encoding a predicted protein, CbFAD7 (439 aa, 50.2 kDa, pI = 7.89), having the highest identity (85%) to *Brassica napus* BnFAD7 (FJ985690). *CbFAD8* contains an ORF encoding a predicted protein, CbFAD8 (397 aa, 45.6 kDa, pI = 8.92), having the highest identity (84%) to *Arabidopsis* AtFAD8 (NM120640).

**Figure 1:**
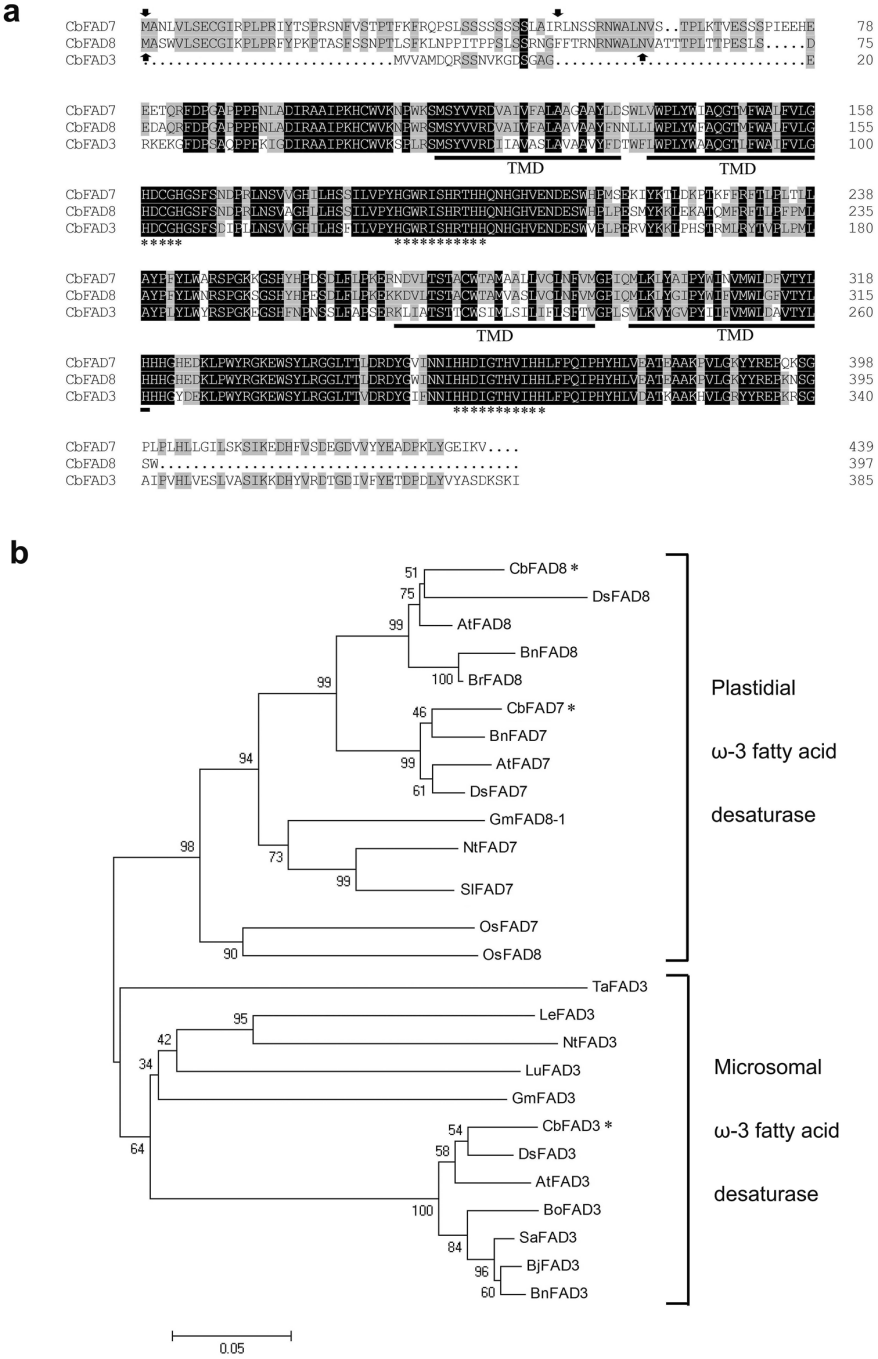
Analysis of the deduced amino acid sequences of *C. bungeana* ω-3 FADs. (**a**) Sequence comparison of CbFAD3, CbFAD7 and CbFAD8. Identical and similar residues are shown on a background of black and gray, respectively. The sequences of the putative chloroplast transit peptides of CbFAD7 and CbFAD8 are arrowed. The three conserved histidine clusters are indicated by asterisks, and the four transmembrane domains (TMD) are underlined. (**b**) Phylogenetic tree analysis of CbFAD3, CbFAD7 and CbFAD8. The positions of ω-3 CbFADs are asterisked. The accession number of different ω-3 FADs included in this analysis: *Arabidopsis thaliana* AtFAD3 (NP180559), *Brassica juncea* BjFAD3 (ADJ58019), *Brassica napus* BnFAD3 (NP001302640), *Brassica oleracea* BoFAD3 (AGH20189), *Chorispora bungeana* CbFAD3 (KM591203), *Chorispora bungeana* CbFAD7 (KY069282), *Chorispora bungeana* CbFAD8 (KY069283), *Descurainia sophia* DsFAD3 (ABK91879), *Glycine max* GmFAD3 (NP001237507), *Lycopersicon esculentum* LeFAD3 (ABX24525), *Linum usitatissimum* LuFAD3 (AFJ53089), *Nicotiana tabacum* NtFAD3 (P48626), *Sinapis alba* SaFAD3 (AHA05997), *Triticum aestivum* TaFAD3 (BAA28358), *Arabidopsis thaliana* AtFAD7 (P46310), *Brassica napus* BnFAD7 (ACS26170), *Descurainia sophia* DsFAD7 (ABS86961), *Nicotiana tabacum* NtFAD7 (D79979), *Solanum lycopersicum* SlFAD7 (NP001234592), *Oryza sativa* OsFAD7 (BAE79783), *Arabidopsis thaliana* AtFAD8 (P48622), *Brassica napus* BnFAD8 (NP001302644), *Brassica rapa* BrFAD8 (AAW78909), *Descurainia sophia* DsFAD8 (ABK91881), *Glycine max* GmFAD8-1 (NP001238609), *Oryza sativa* OsFAD8 (BAE79784).

Using the targetP prediction tool, two chloroplast targeting peptide of 50 and 60 aa were found in the N-terminal of the deduced CbFAD7 and CbFAD8 (Fig. 1a), respectively, predicting the subcellular localization of the proteins in chloroplasts. Amino acid alignment (Fig. 1a) showed that both of the deduced proteins contain three conserved histidine clusters (HDGCH, HXXXXXHRTHH and HHXXXXHVIHH) and four transmembrane domains (TMD), suggesting that they are chloroplast membrane-bound ω-3 FADs. The phylogenetic analysis (Fig. 1b) displayed that CbFAD7 (ARL62096) and CbFAD8 (ARL62097) were positioned in the group corresponding to plastidial ω-3 FADs, providing further evidence that *CbFAD7* and *CbFAD8* encode plastidial ω-3 FADs.

### The functionality of *CbFAD7* and *CbFAD8* were verified in *Arabidopsis* mutant

**Figure 2:**
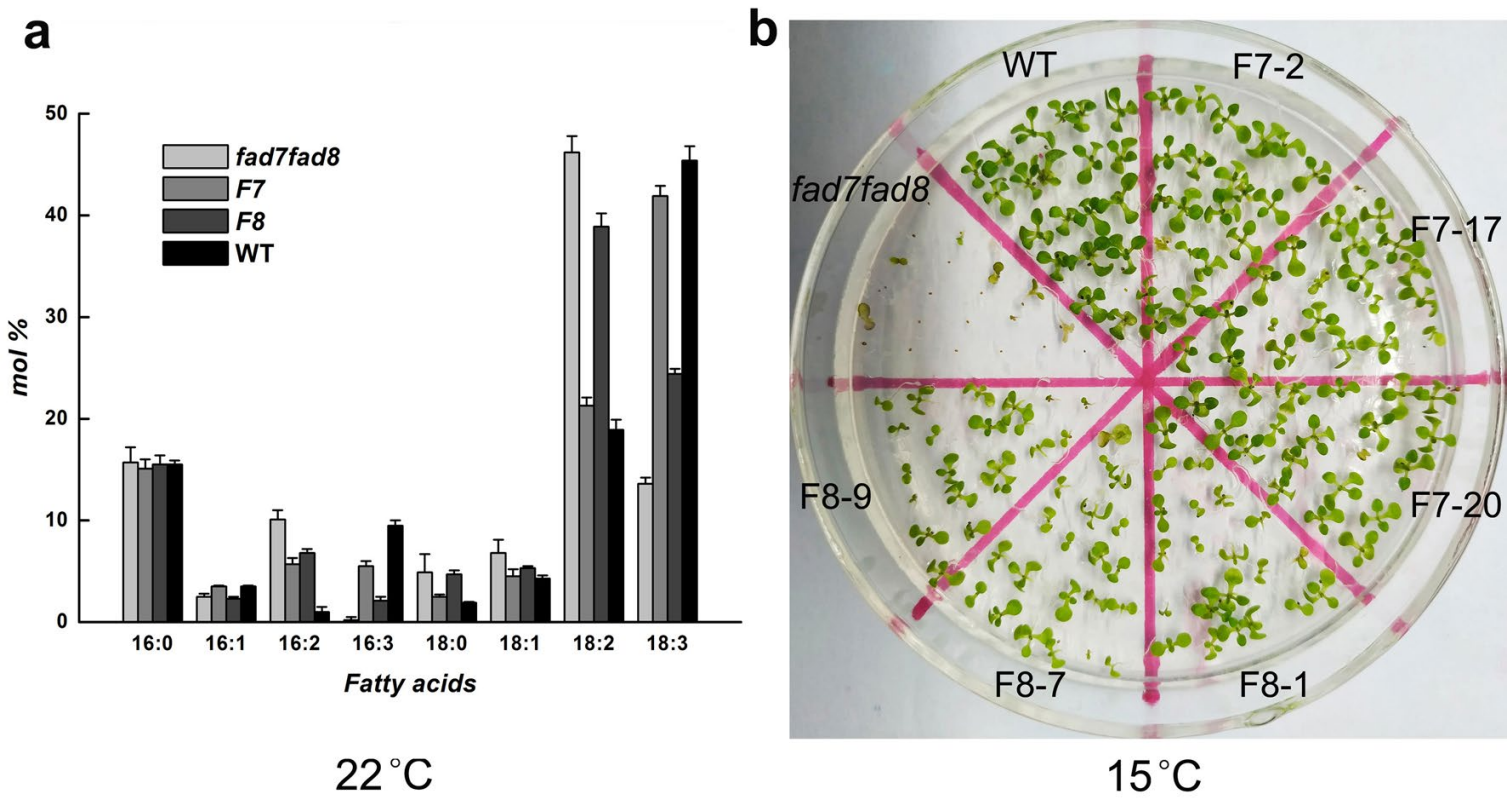
Functionality of *CbFAD7* and *CbFAD8* were verified in *Arabidopsis* mutant. (**a**) Comparison of total leaf fatty acids between *Arabidopsis* lines under normal conditions. (**b**) Comparison of low-temperature germination between *Arabidopsis* lines. WT means wild type (Col-0); *fad7fad8* means double *fad7fad8* mutant; F7 means *CbFAD7*-complemented mutant; F8 means *CbFAD8*-complemented mutant. Each value represents the mean ± SE of five replicates.

To verify the functionality of *CbFAD7* and *CbFAD8*, the ORF of the two genes were expressed in double *fad7fad8* mutants under the CaMV 35S promoter of pBI121 vector, respectively. The fatty acids of leaf lipids showed that though the C18:3 contents in the complemented mutants F7 and F8 were still lower than that in WT plants, they were markedly higher than that in *fad7fad8* mutants (Fig. 2a). Being exposed to 15 °C, the germination rates of F7 and F8 seeds were significantly higher than that of *fad7fad8* mutant seeds, and close to that of WT seeds (Fig. 2b). These data confirmed that *CbFAD7* and *CbFAD8* were functional plastical ω-3 *FAD* genes.

### The tissue-specific and cold-responsive expressions of ω-3 *CbFAD* genes in *C. bungeana*

**Figure 3:**
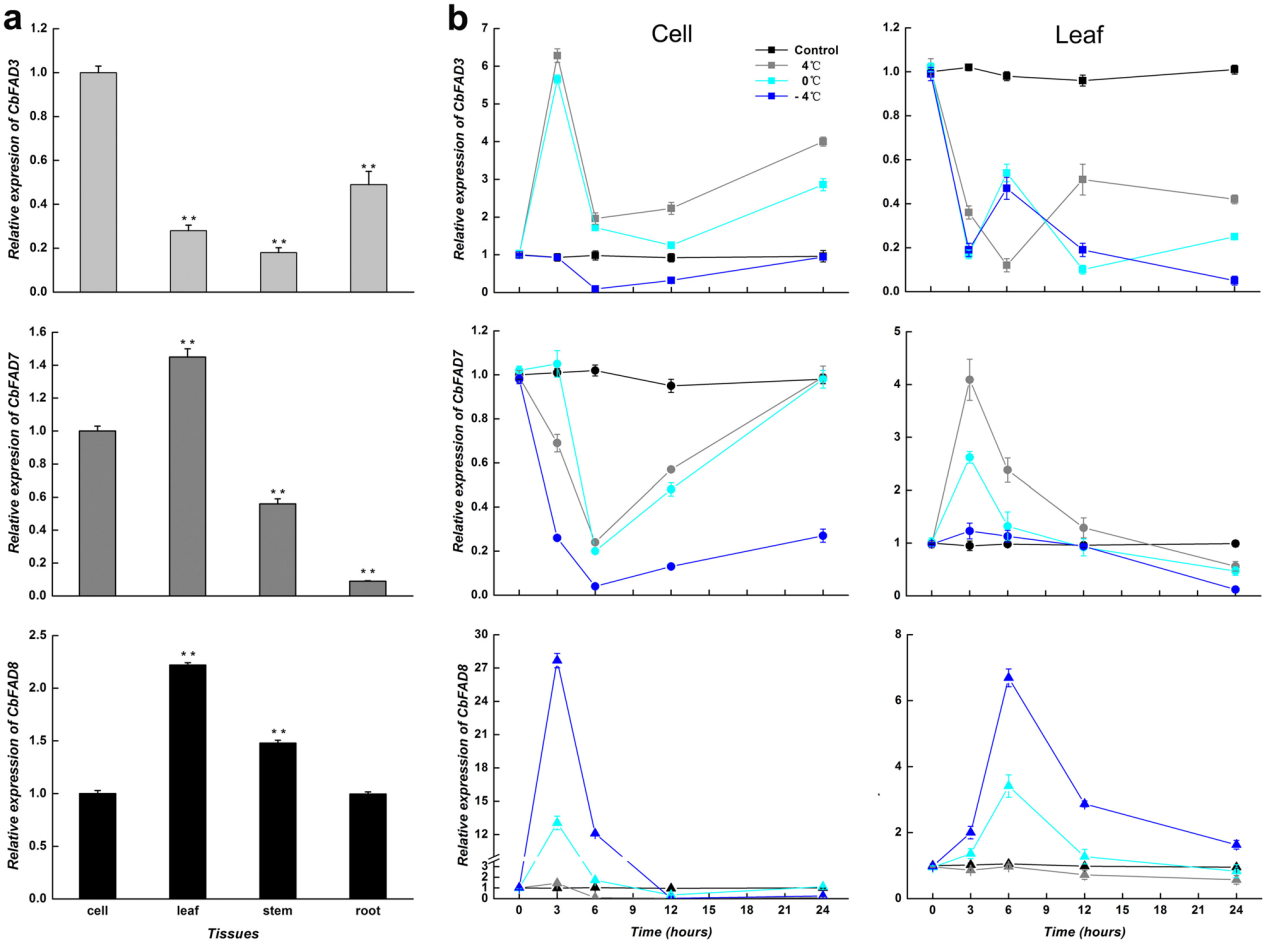
Expression patterns of ω-3 *CbFAD* genes in *C. bungeana.* (**a**) Tissue-specific expressions of *CbFAD3*, *CbFAD7* and *CbFAD8*. Data were presented as relative expression ratios being compared with the expression levels of corresponding genes in suspension-cultured cells, which were set at a value of 1. (**b**) Cold-responsive expressions of *CbFAD3*, *CbFAD7* and *CbFAD8* in suspension-cultured cells and the leaves from regenerated plants. Data were presented as relative expression ratios being compared with the expression levels of corresponding genes before treatment (0 h), which were set at a value of 1. Each value represents the mean ± SE of three replicates. The relative expressions of *CbFAD3*, *CbFAD7* and *CbFAD8* are indicated by square, circle and triangle, respectively.

The expression profiles of plastical and microsomal ω-3 *CbFAD* genes were analyzed in the suspension-cultured cells and the regenerated plants of *C. bungeana* (Fig. 3a). *CbFAD7* and *CbFAD8* have the highest expression in leaves and the lowest expression in roots, showing the characteristic of plastidial ω-3 *FAD* genes*. CbFAD3* were mostly expressed in suspension-cultured cells, and lowly expressed in stems, exhibiting the feature of microsomal ω-3 *FAD* genes.

Considering the highest expression of microsomal and plastidial ω-3 *CbFAD* genes, the cold-responsive expressions of them were detected in suspension-cultured cells and the leaves from regenerated plants (Fig. 3b). In suspension-cultured cells, the expression of *CbFAD3* was increased at 4 (6.3-fold) and 0 °C (5.7-fold), while that of *CbFAD8* was increased at 0 (13.1-fold) and − 4 °C (27.7-fold). The increases in *CbFAD3* and *CbFAD8* mRNA all peaked at being treated for 3 h, and were accompanied by a decrease in *CbFAD7* mRNA at different low temperatures. In *C. bungeana* leaves, the increase in *CbFAD7* mRNA was found at 4 (4.1-fold) and 0 °C (2.6-fold), and the induction of *CbFAD8* expression also occurred at 0 (3.4-fold) and − 4 °C (6.7-fold), like that found in cultured cells. Moreover, the cold-induced expression of *CbFAD7* and *CbFAD8* peaked at being treated for 3 and 6 h, respectively, which were accompanied by a decrease in *CbFAD3* mRNA at tested low temperatures. In summary, the expression of ω-3 *CbFAD* genes presented a non-redundant pattern in response to low temperatures.

### The hormone- and inhibitor-responsive expressions of ω-3 *CbFAD* genes in *C. bungeana*

**Figure 4:**
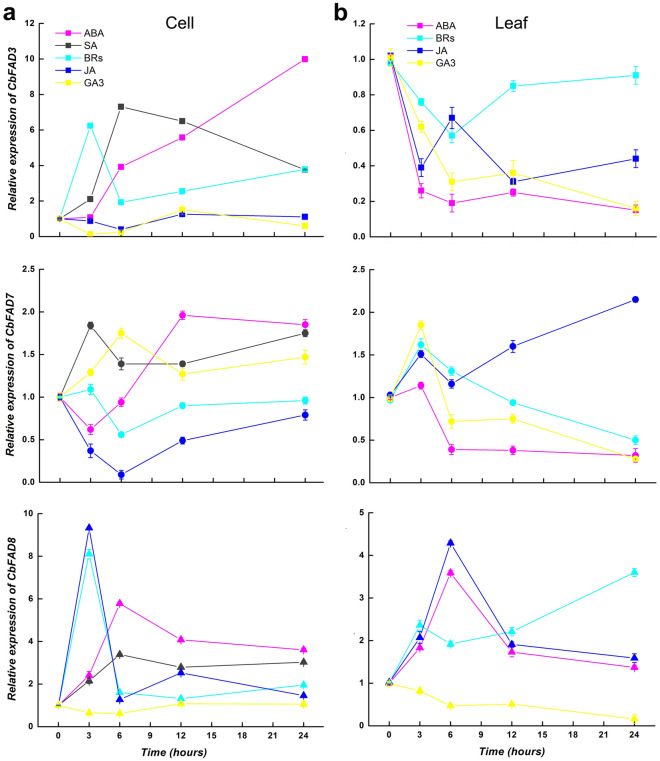
Hormone-responsive expressions of ω-3 *CbFAD* genes in *C. bungeana.* (**a**) Expression patterns of *CbFAD3*, *CbFAD7* and *CbFAD8* in suspension-cultured cells. (**b**) Expression patterns of *CbFAD3*, *CbFAD7* and *CbFAD8* in the leaves from regenerated plants. Data were presented as relative expression ratios being compared with the expression levels of corresponding genes before treatment (0 h), which were set at a value of 1. Each value represents the mean ± SE of three replicates. The relative expressions of *CbFAD3*, *CbFAD7* and *CbFAD8* are indicated by square, circle and triangle, respectively.

To be in line with the cold-responsive experiments, the hormone-responsive experiments on ω-3 *CbFAD* genes were also studied in suspension-cultured cells and the leaves from regenerated plants. Data (Figs. 3b and 4) showed that though each of the tested hormones could affect the expression of ω-3 *CbFAD* genes, only the changes caused by certain hormones were similar to those induced by low temperatures, considering change trend and peak time. In respect to cell ω-3 *CbFAD* genes, the similar changes were all brought by JA and BRs. However, as for leaf ω-3 *CbFAD* genes, the regulation were more complex: the corresponding down-regulation of *CbFAD3* expression were caused by JA, BRs, ABA and GA3; the comparable increases in *CbFAD7* and *CbFAD8* mRNA were induced by BRs and GA3 (1.6- and 1.9-fold, peaked at 3 h) as well as JA and ABA (4.3- and 3.6-fold, peaked at 6 h), respectively. The Pearson correlations between the cold- and the hormone-responsive expressions of ω-3 *CbFAD* genes (Tables 1 and 2) indicated that JA and BRs may participate in the low-temperature-responsive expressions of these genes in both suspension-cultured cells and plant leaves, while ABA and GA3 may only take part in the low-temperature-induced regulation in leaves.

**Table 1:** Pearson correlation coefficients (two-tailed) between the cold-responsive and the hormone-regulated expressions of ω-3 CbFAD genes in *C. bungeana* cells.

Gene name	Temperature (℃)	SA	BRs	JA	ABA	GA3
*CbFAD3*	4	− 0.26	0.99***	0.07	0.04	− 0.55
	0	− 0.35	0.97***	− 0.07	− 0.16	− 0.67
	− 4	− 0.94	0.35	0.51*	− 0.09	0.10
*CbFAD7*	4	− 0.02	0.78**	0.94**	0.22	− 0.65
	0	0.23	0.91***	0.72**	− 0.14	− 0.64
	− 4	− 0.61	0.48	0.82***	− 0.23	− 0.78
*CbFAD8*	4	− 0.73	0.72**	0.71**	− 0.75	− 0.34
	0	− 0.16	0.99***	0.97***	− 0.26	− 0.60
	− 4	0.02	0.91***	0.87***	0.00	− 0.83

Three replications for each sample, *n* = 5 × 3. Significant positive correlation (*R* > 0.50, *P* < 0.05) is indicated in bold. *P < 0.05, **P < 0.01, ***P < 0.001.

**Table 2:** Pearson correlation coefficients (two-tailed) between the cold-responsive and the hormone-regulated expressions of ω-3 CbFAD genes in *C. bungeana* leaves.

Gene name	Temperature (℃)	BRs	JA	ABA	GA3
*CbFAD3*	4	0.85***	0.60*	0.90***	0.78**
	0	0.23	0.99***	0.86***	0.71**
	− 4	0.22	0.95***	0.91***	0.82***
*CbFAD7*	4	0.94***	− 0.24	0.47	0.86***
	0	0.93***	− 0.29	0.49	0.95***
	− 4	0.90***	− 0.77	0.46	0.74**
*CbFAD8*	4	− 0.76	0.36	0.39	0.69*
	0	− 0.21	0.97***	0.97***	− 0.11
	− 4	− 0.08	0.98***	0.98***	− 0.29

Three replications for each sample, *n* = 5 × 3. Significant positive correlation (*R* > 0.50, *P* < 0.05) is indicated in bold. *P < 0.05, **P < 0.01, ***P < 0.001.

**Figure 5:**
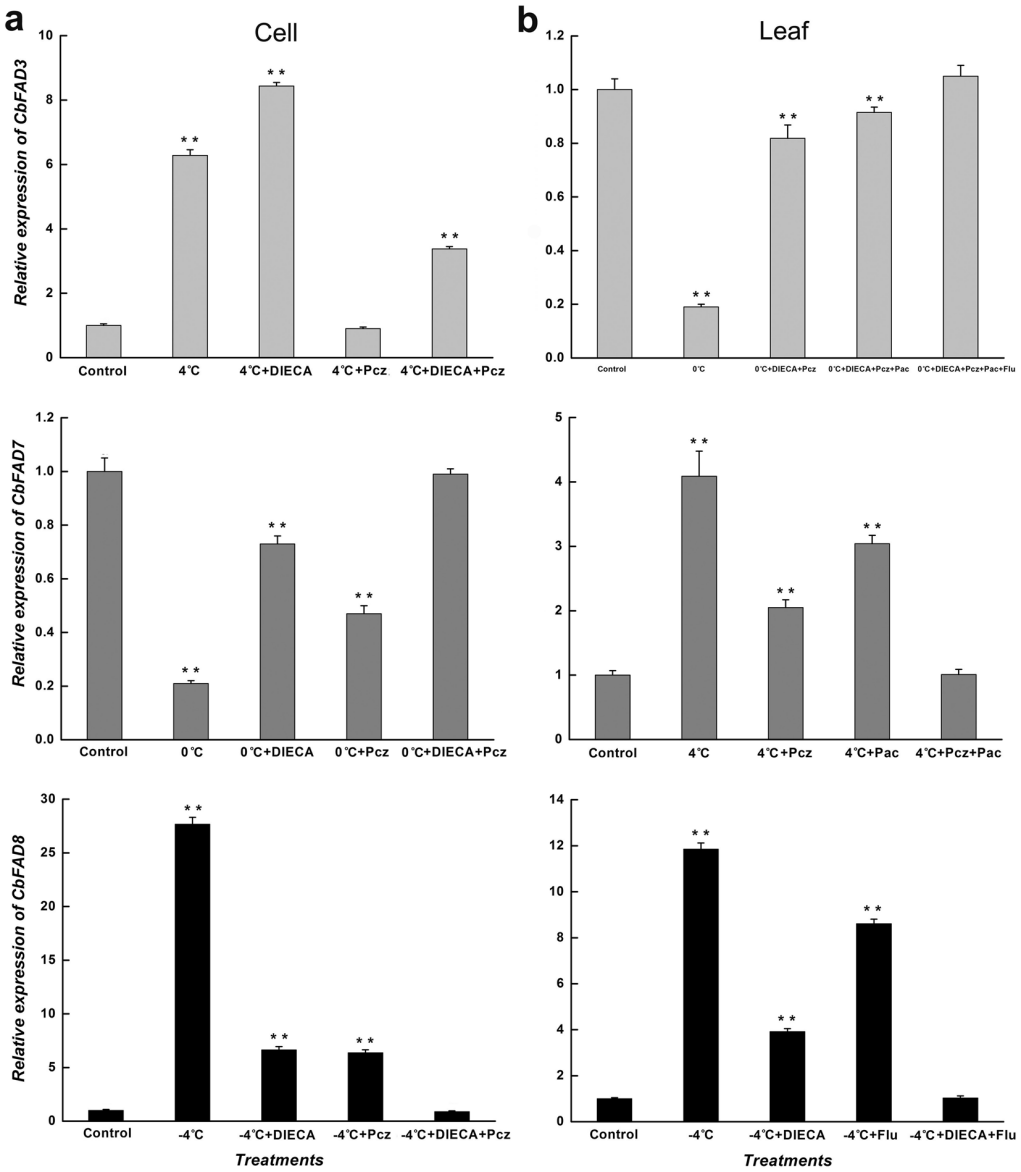
Hormone inhibitors affected the cold-responsive expressions of ω-3 *CbFAD* genes in *C. bungeana.* (**a**) Expression levels of *CbFAD3*, *CbFAD7* and *CbFAD8* in suspension-cultured cells. The expressions of *CbFAD3* and *CbFAD8* were detected at being treated for 3 h, while that of *CbFAD7* was detected at being treated for 6 h. (**b**) Expression levels of *CbFAD3*, *CbFAD7* and *CbFAD8* in the leaves from regenerated plants. The expressions of *CbFAD3* and *CbFAD7* were detected at being treated for 3 h, while that of *CbFAD8* was detected at being treated for 6 h. Data were presented as relative expression ratios being compared with the expression levels of corresponding genes under normal conditions (Control), which were set at a value of 1. Each value represents the mean ± SE of three replicates.

To further confirm the participation of these hormones, the corresponding inhibitors were used. As showed by Fig. 5, in suspension-cultured cells, the chilling-induced increase in *CbFAD3* mRNA was totally inhibited by Pcz (a synthetic inhibitor of BRs), but the inhibition could be partly relieved by DIECA (a synthetic inhibitor of JA); the cold-inhibited expression of *CbFAD7* was partially eliminated by Pcz (32.9%) or DIECA (65.8%), and was completely eliminated by the combination of them; conversely, the cold-induced expression of *CbFAD8* was mostly inhibited by Pcz (78.9%) or DIECA (79.9%), and was entirely inhibited by the cooperation of them. In *C. bungeana* leaves, the decrease in *CbFAD3* mRNA caused by low temperatures was absolutely eliminated by the synergism of Pcz, DIECA, Pac (a synthetic inhibitor of GA3) and Flu (a synthetic inhibitor of ABA), and the synergistic effect of Pcz and DIECA play a major role (79.0%); Although the chilling induction of *CbFAD7* expression could be suppressed by Pcz (66.0%) or Pac (33.9%) to some extent, the complete suppression need the joint action of them; likewise, the cold induction of *CbFAD8* expression was incompletely inhibited by DIECA (73.1%) or Flu (29.9%), but was thoroughly inhibited by the combined effect of them.

Altogether, these results suggested that JA and BRs may result in the opposite changes in cell *CbFAD3* expression, while the synergism of them may bring cell *CbFAD7* inhibition and cell *CbFAD8* induction, except the high level of BRs (Supplementary Fig. S2). In *C. bungeana* leaves, JA and BRs may lead to decrease in *CbFAD3* mRNA with the help of ABA and GA3, and the combined effect of BRs and GA3 may active the chilling-responsive induction of *CbFAD7* expression, while the joint action of JA and ABA may trigger the cold-responsive induction of *CbFAD8* expression.

### The level of related phytohormones in *C. bungeana* during low-temperature treatments

**Figure 6:**
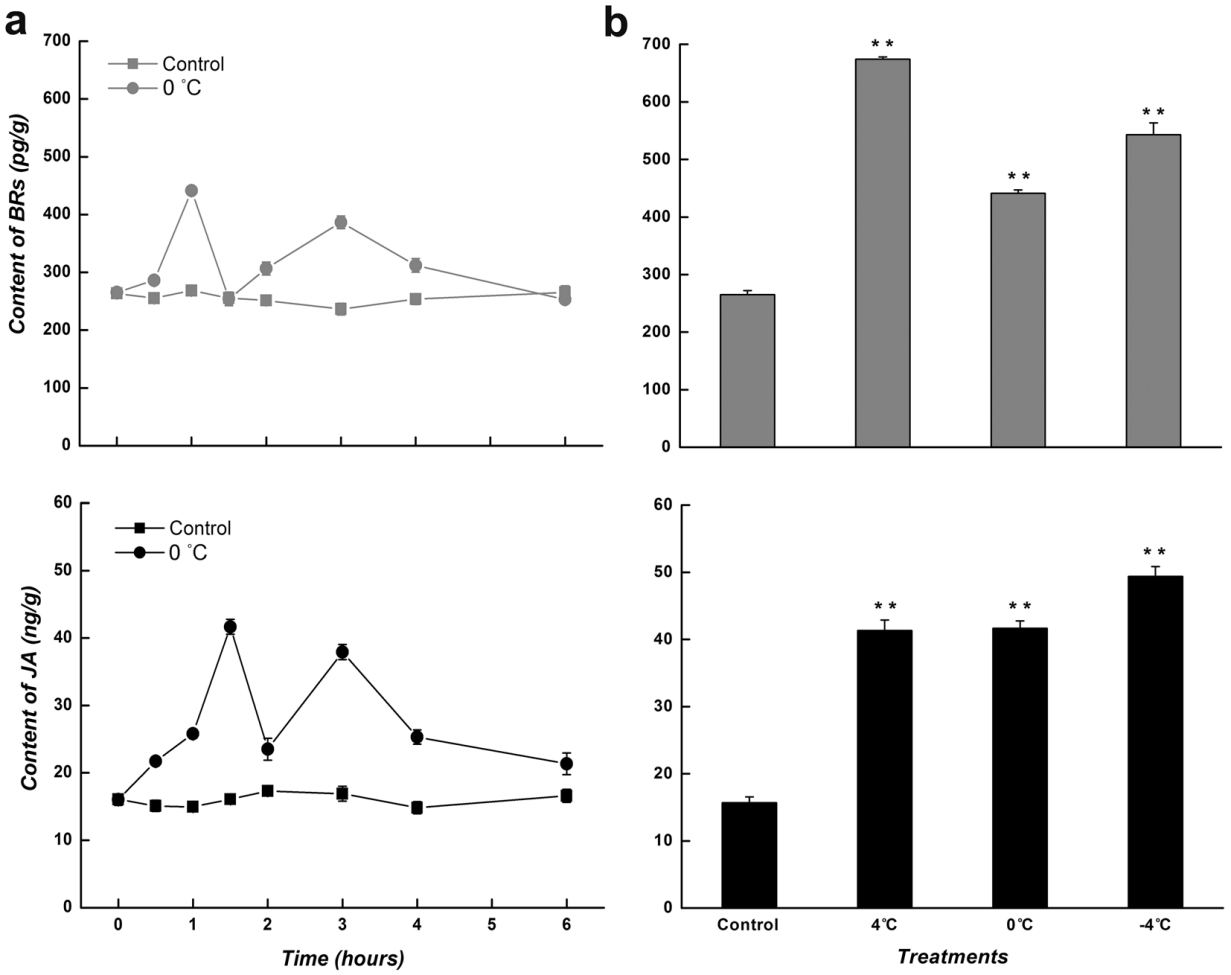
Pytohormone analysis in the suspension-cultured cells of *C. bungeana* at low temperatures. (**a**) Changes in the level of BRs and JA at 0 °C (**b**) Levels of BRs and JA at different low temperatures (4, 0 and − 4 °C). The data of BRs was detected at being treated for 1 h, and that of JA was detected at being treated for 1.5 h. The corresponding data at the same time point under normal conditions was taken as the control. Each value represents the mean ± SE of five replicates.

**Figure 7:**
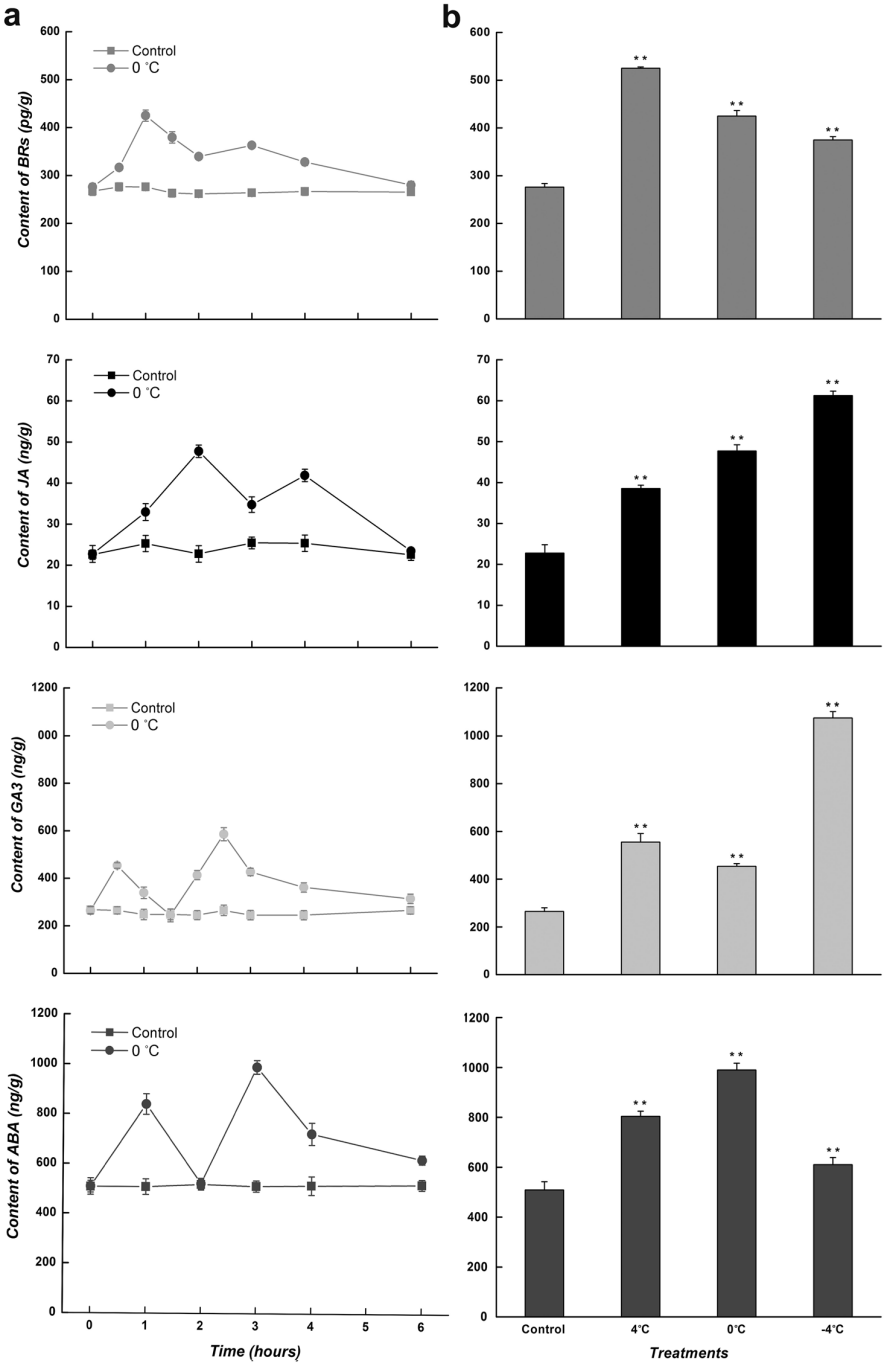
Pytohormone analysis in the leaves from *C. bungeana* regenerated plants at low temperatures. (**a**) Changes in the level of BRs, JA, GA3 and ABA at 0 °C. (**b**) Levels of BRs, JA, GA3 and ABA at different low temperatures (4, 0 and − 4 °C). The data of BRs and ABA were detected at being treated for 1 h, and those of JA and GA3 were detected at being treated for 2 and 0.5 h, respectively. The corresponding data at the same time point under normal conditions was taken as the control. Each value represents the mean ± SE of five replicates.

To provide more evidence to the hormone-regulated cold response, the level of related phytohormones were detected in suspension-cultured cells and the leaves from regenerated plants at different low temperatures. Being exposed to 0 °C, the level of tested pyhtohormones presented a rapid and two-peaks increase in both analysis systems, however, the peak time and the peak value of them were various (Figs. 6a and 7a). In cultured cells, the level of BRs peaked at being treated for 1 (1.7-fold) and 3 h (1.5-fold), while that of JA peaked at being treated for 1.5 (2.6-fold) and 3 h (2.4-fold). Though the accumulations of BRs and JA in leaves were similar to those in cultured cells, the peak time of JA (2 and 4 h) was a little later. The change trends of GA3 (1.8- and 2.4-fold) and ABA (1.6- and 2.0-fold) resembled each other, but the peak time of GA3 (0.5 and 2.5 h) was half an hour earlier than that of ABA. Overall, the phytohormone increases induced by low temperatures, notably the first peak, preceded the temperature-responsive expression changes in corresponding ω-3 *CbFAD* genes; furthermore, the level of synergistic hormones, such as JA and ABA, or BRs and GA3, reached the peak value at staggered times to avoid redundant effect.

Although the phytohormone increases could be induced by different low temperatures, the increments were varied with temperature (Figs. 6b and 7b). In suspension-cultured cells, the highest level of BRs and JA appeared at 4 (2.6-fold) and − 4 °C (3.1-fold), respectively. In *C. bungeana* leaves, the increased level of BRs was decreased with decreasing temperature, while that of JA was increased; meanwhile, the increase in the level of GA3 and ABA at 0 and − 4 °C presented an opposite trend. Together, the hormone increase were consistent with the dynamic expressions of corresponding ω-3 *CbFAD* genes at different low temperatures; moreover, the increased level of antagonistic hormones, for example GA3 and ABA, showed a reverse trend during temperature variation, which may be due to the trade-offs between plant growth and cold stress response.

### The level of TAs in *C. bungeana* during low-temperature and hormone-inhibitor treatments

**Figure 8:**
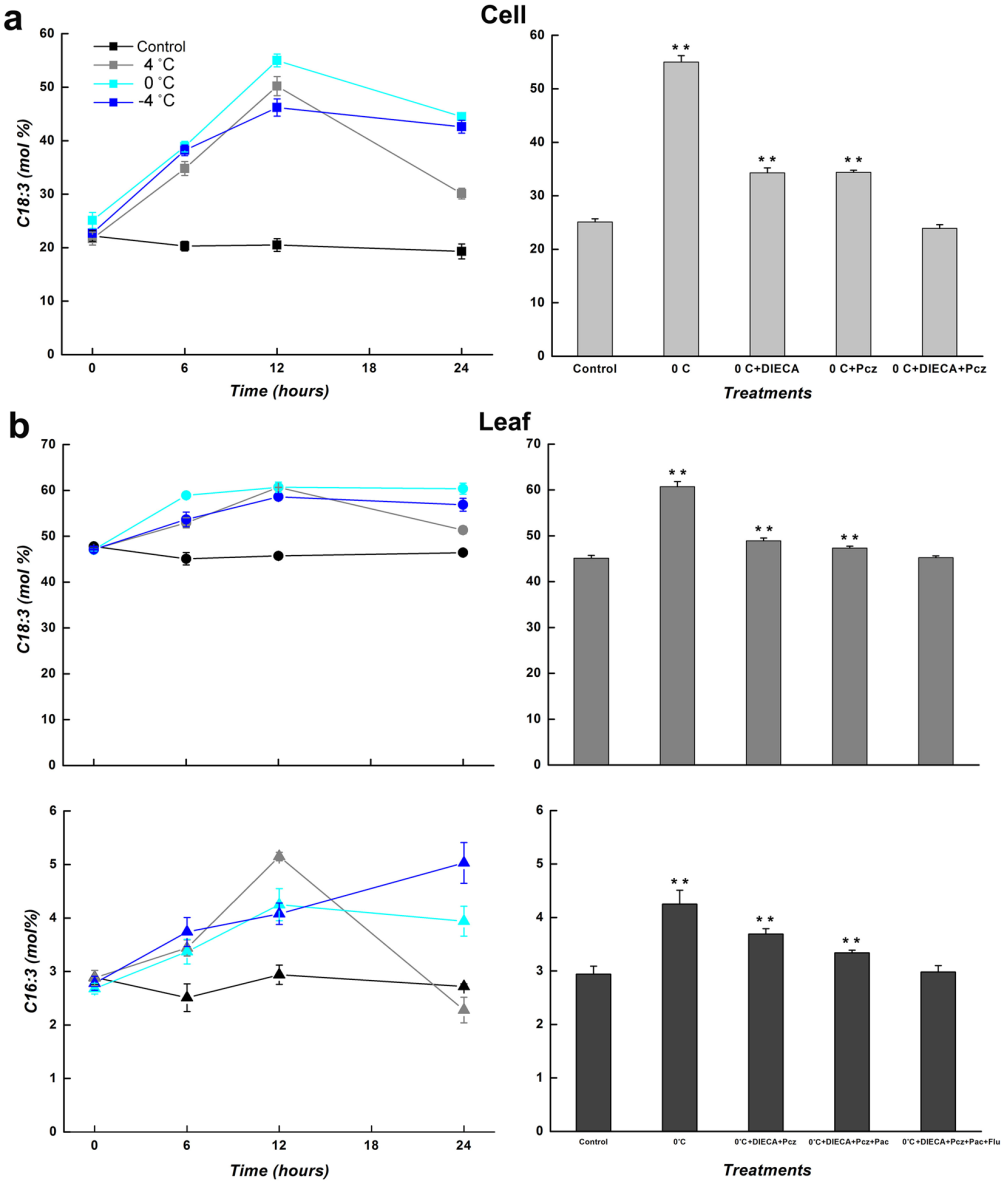
Levels of TAs in *C. bungeana* under low-temperature and hormone-inhibition treatments. (**a**) Levels of C18:3 in the total lipids of suspension-cultured cells. (**b**) Levels of C18:3 and C16:3 in the total lipids of the leaves from regenerated plants. The data of inhibition treatments were detected at being treated for 12 h. The corresponding data at the same time point under normal conditions was taken as the control. Each value represents the mean ± SE of five replicates. The content of C18:3 in cell lipids and leave lipids as well as the content of C16:3 in leave lipids are indicated by square, circle and triangle, respectively.

Considering that the temperature-responsive expression of ω-3 *CbFAD* genes may affect the accumulation of TAs, the content of C18:3 and C16:3 were tested in the total lipids of suspension-cultured cells and the leaves from regenerated plants at different low temperatures. In the absence of well-developed chloroplasts, no C16:3 was detected in the lipids from cultured cells. In both analysis systems (Fig. 8), the accumulation of C18:3 was obviously induced by tested low temperatures and reached the maximum at being treated for 12 h: the content of C18:3 in cell lipids increased from about 20.6% to 46.2–55.0% of total fatty acids, while that in leaf lipids increased from about 46.3% to 58.6–60.7% of total fatty acids. Similarly, the content of C16:3 in leaf lipids increased from about 2.8% to 4.3–5.2% of total fatty acids, and reached the maximum at being treated for 12 (4, 0 °C) or 24 h (− 4 °C). The results revealed that at low temperatures, the increases in TAs agreed with the expression changes in ω-3 *CbFAD* genes with a time lag.

To further confirm that the phytohormones affect the accumulation of TAs through gene regulation, the hormone-inhibitor treatments were performed at 0 °C (Fig. 8). In suspension-cultured cells, the cold-induced accumulation of C18:3 was completely inhibited by the synergism of DIECA and Pcz, but partially inhibited by either of them. In *C. bungeana* leaves, the cold-induced accumulation of C18:3 and C16:3 were both totally suppressed by the synergistic effect of DIECA, Pcz, Pac and Flu, but partly suppressed by either of them. These results were in accord with the inhibitor-responsive expression of ω-3 *CbFAD* genes, and verified that with or without the help of ABA and GA3 in distinct tissues, JA and BRs participated in maintaining appropriate level of TAs in *C. bungeana* through regulating ω-3 *CbFAD* genes in response to low temperatures.

## Discussion

This work analyzed the behavior of plastidial and microsomal ω-3 FADs in *C. bungeana* at the level of gene expression and fatty acid content, to determine how low temperature affected the contribution of ω-3 FADs to the synthesis of TAs, especially C18:3. From the result, it can be seen that low temperatures resulted in significant transcriptional changes on each ω-3 CbFAD. Among these changes, the sharply increased *CbFAD8* mRNA induced only by severe low temperatures (notably the subzero temperature) was observed both in the suspension-cultured cells and in the leaves from regenerated plants (Fig. 3b). Considering the difference between the two analysis systems, this common phenomenon shows the critical role of CbFAD8 in the synthesis of TAs under freezing and subfreezing temperatures, which is different with the way most plant species do. In most plant species^12–15,17,18,20,42^, the transcript level of *FAD8* was increased in response to chilling temperatures ranging from 4 to 15 °C. FAD8 was originally identified as a cold-specific desaturase by phenotypic analysis of a *fad3fad7* double mutant from *Arabidopsis*^43^ that was capable of producing TAs only at low temperatures (15 °C). Given that FAD8 plays an important role in the biosynthesis of plastid TAs^44,45^ required for the correct biogenesis and maintenance of chloroplasts^46^ as well as the recovery from photo inhibition at low temperatures^47,48^, the induction of *FAD8* expression shows a common choice of plants, namely protecting chloroplasts, particularly photosynthesis. In *C. bungeana*, the induction of *CbFAD8* occurred at or below 0 °C may be due to its original membrane lipid unsaturation of chloroplasts brought by FAD8 is higher than that in most other plants, which can help the chloroplasts to get through chilling temperatures. This may also explain why the cold resistance of *C. bungeana* is much higher than that of normal plants. Of course, it still needs further researches.

In response to chilling temperatures, the expression of *CbFAD3* and *CbFAD7* presented a tissue-specific profile (Fig. 3b). The *CbFAD3* mRNA was increased in suspension-cultured cells and decreased in plant leaves, while the expression profile of *CbFAD7* was just the opposite. Combined with previous studies, we found that even in the same tissue under similar temperature conditions, the low-temperature-induced expression of *FAD3* and *FAD7* were varied with plant species^7,12–14,16,17,49–51^, which means some observations are consistent with ours but the others are not. Although we don’t know why the contributions of microsomal and plastidial desaturases differ among plant species, it is not surprising that the contributions of CbFAD3 and CbFAD7 in *C. bungeana* were proportional to their transcript abundance in corresponding tissues (Fig. 3). Furthermore, this expression profile suggests that CbFAD3 would be more important in *C. bungeana* cultured cells while CbFAD7 might contribute to TAs production in *C. bungeana* leaves under chilling temperatures.

When the temperature was further decreased, the increase in *CbFAD3* or *CbFAD7* mRNA was reduced and even turned into a decrease at -4 °C, which formed a non-redundant complementation with the gradual increase in *CbFAD8* mRNA (Fig. 3b). The coordination of *CbFAD3* and *CbFAD8* indicates the distinct TA needs of *C. bungeana* cells at different low temperatures, for FAD3 affecting total TA levels and FAD8 affecting plastid TA levels^44,45^. The cooperation of *FAD7* and *FAD8* was not only found in *C. bungeana* leaves, but also found in *Arabidopsis* leaves^18^ at different temperatures (8, 22 and 30 °C). Although both FAD7 and FAD8 affect the synthesis of TAs in chloroplastic lipids, the lipid specificity of them are not the same^27^: AtFAD7 prefers galactolipids, which are the major chloroplast lipids with higher TA content; AtFAD8 likes phosphatidylglycerol^18^, which has a specific role in the stability of photosynthetic complexes^52^. Hence, the trade-off between *CbFAD7* and *CbFAD8* in leaves reflects the strategy to maintain photosynthetic activity and stability during temperature variation. Nevertheless, the non-redundant expression of ω-3 *CbFAD* genes maintained appropriate level of TAs, especially C18:3, in response to low temperatures (Fig. 8a).

Through a series of verification, including exogenous hormone application (Fig. 4), correlation analysis (Tables 1 and 2), inhibitor treatments (Figs. 5 and 8) and phytohormone detection (Figs. 6 and 7), we found that the low-temperature-responsive expression of ω-3 *CbFAD* genes were regulated by certain phytohormones, notably JA and BRs. JA was mainly responsible for *FAD8* induction under freezing/subfreezing temperatures, with the tissue-specific assistance of BRs or ABA; BRs was in charge of the induction of *CbFAD*3 or *CbFAD7* expression at chilling temperatures, with or without the help of GA3 in distinct tissues. Moreover, both JA and BRs took part in the inhibition of corresponding ω-3 *CbFAD* expression in response to low temperatures, with or without the participation of ABA and GA3 according to tissue specificity. These data not only provide new insights into our previous findings^17^ that JA partially mediates the chilling-induced transcription of ω-3 *FAD* genes in *Arabidopsis*, but also agree with that JA may act as a core signal by interacting with other phytohormones to regulate the balance between plant growth and stress response^53^. As we know (Supplemetary Fig. S3), ABA may interact synergistically with JA signaling to regulate the expression of cold-responsive genes^54–56^; BRs acts in synergism or antagonism with JA, depending on BRs’ concentration in response to stress^53^; GA usually inhibits JA signaling, but in some cases, the synergistic effect of them is also exist^53,54,56^. In this study, the interactions between JA and the other tested hormones are in line with the reported findings. Notably, the joint action of JA and ABA regulated the cold-induced expression of leaf *CbFAD8*, and the combined effect of JA and BRs triggered the induction of *CbFAD8* expression in cultured cells. Our previous study in *Arabidopsis*^17^, which confirmed the participation of JA in the chilling-induced expression of ω-3 *FAD* genes, also implied the existence of JA’s partner. Besides that, a recent study reported that in *Arabidopsis* leaves^27^, AtFAD8 protein levels were increased upon cold or JA exposure, but did not respond to ABA. All these data prove the fact that though the partner of JA signaling may vary with tissues and plant species, JA did regulate the low temperature induction of *FAD8* expression in both *C. bungeana* (cryophyte) and *Arabidopsis* (modal plant), which may be common in most plant species.

In *C. bungeana*, BRs is another important pyhtohormone involved in the low-temperature regulation of ω-3 *FAD* genes. It is confirmed that BRs can improve frost tolerance by promoting GA biosynthesis and interplaying with GA at the signaling level^57^ (Supplemetary Fig. S3), which supports the observation that the low temperature response of *CbFAD3* and *CbFAD7* in *C. bungeana* leaves was mediated by the cooperation of BRs and GA3 (Fig. 5b). Recent studies also demonstrated that BRs may participate in drought or cold stress acclimation by three interconnected mechanisms, one of which is in communication with ABA signaling^58^ (Supplemetary Fig. S3). This may explain the synergistic effect of BRs and ABA on the down-regulation of leaf *CbFAD3* expression during low temperature exposure (Fig. 5b). To date, there is no report about the participation of BRs in the expression of ω-3 *FAD* genes in response to low temperatures, so we cannot predict that BRs also regulates the low temperature induction of *FAD3* or *FAD7* expression in other plant species before further research.

It is worth noting that the interaction between different phytohormones demonstrates that each ω-3 *CbFAD* gene can respond to multiple hormones (Figs. 4, 5, 6, 7 and 8; Tables 1 and 2). A similar phenomenon was found in *Arabidopsis*, which showed the expression of *AtFAD3* was regulated through the synergistic and antagonistic interactions of auxin, cytokinin and ABA during plant development^59^. These results reflect the existence of various promoter cis-elements combined with different transcription factors (TFs) from corresponding hormone signaling. A G-box-like motif required by JA-responsive expression was first found in the promoter of *AtFAD7* from *Arabidopsis* roots^60^. After that, the SA- and ABA-responsive elements were found in the promoter of cabbage *FAD8*^61^. Recently, the analysis on *AmFAD7* and *AmFAD8* in *Ammopiptanthus mongolicus* confirmed that both of the promoters contain the elements for the response to ABA, JA, GA and MeJA^62^. Moreover, the multiple ABA-responsive elements were found in the promoter of microalgae ω-3 *FAD* genes^63^. All the findings support the idea that ω-3 *FAD* genes can be directly regulated by more than one hormone.

As another necessary factor for the hormone regulation, TFs have been studied at recent years. On the one hand, some TFs related to ω-3 *FAD* expression were verified: for example, the expression of *FAD3* was up-regulated by bZIP67 in *Arabidopsis* seeds^64^, but down-regulated by MaMYB4 in banana fruits^65^ or by HD in soybean^66^; WIPK was involved in wound-responsive expression of *AtFAD7* gene in transgenic tobaccos^67^. On the other hand, it is confirmed in *Arabidopsis* that some TFs (such as WRKYs, MYCs, bHLHs and ICEs) occurred at the downstream of cold-induced JA signaling^54,56^, while some others (for example bZIPs) belong to the cold-responsive BR and/or ABA signaling pathways^57,68^. Additionally, a recent study in a mutant of *Pyrus bretschneideri Rehd* found that MYB1R1 and MYC2 regulate ω-3 FADs involved in ABA-mediated suberization^69^.

Though all the relevant information are not quite complete, if we piece them together, it is not difficult to speculate that through the common or distinct TFs combined with corresponding promoter elements, JA and BRs as well as ABA and GA3 achieve the synergistic or antagonistic regulation on ω-3 *CbFAD* genes, which result in the non-redundant cooperation on maintaining appropriate level of TAs and then help *C. bungeana* survive in cold environments*.* These results may provide valuable information to agricultural production, for example, multi-hormone application may help crops overcome the influence of low temperatures in the future.

## Methods

### Plant material and experiment treatments

The suspension-cultured cells and the regenerated plants of *C. bungeana* were prepared as described by Shi et al.^3^ and Fu et al.^30^, respectively. Photomixotrophic cultured cells were initiated from wild *C. bungeana* leaves, while regenerated plants were originated from *C. bungeana* cotyledons. Though both of them were propagated under 25 °C with 12 h illuminations (1000 and 4000LX, respectively), the former were germinated in liquid modified MS medium (0.2 mg/L of 2,4-dichlorophenoxyacetic acid, 1-naphthaleneacetic acid, 6-benzylaminopurine and kinetin, respectively), and the latter were grown on gel modified MS medium (0.4 mg/L of gibberellin, 0.6 mg/L of kinetin and 3% glucose instead of sucrose). Regenerated plants having 3–5 cm long roots were used for experiments. *Arabidopsis* seeds of Col-0 (WT), *fad7fad8* mutant (N8036, NASC, UK), and complemented *fad7fad8* mutants (F7 and F8) were cultivated as our previous procedure^17^.

For cold treatments, *C. bungeana* cell suspensions and regenerated plants were exposed to 4, 0  or − 4 °C for 24 h, respectively. For low-temperature germination, *Arabidopsis* seeds planted on MS medium were exposed to 15 °C for 4 weeks. For exogenous hormone treatments, the cell suspensions and regenerated plants were moved to 1/2 MS medium with 100 µM JA, 0.5 µM BRs, 100 µM SA, 100 µM ABA, or 100 µM GA_3_ for 24 h, respectively. For phytohormone inhibitions, the cell suspensions and regenerated plants were moved to 1/2 MS medium with 10 µM sodium diethyldithiocarbamate trihydrate (DIECA), 10 µM propiconazole (Pcz), 10 µM fluricbne (Flu), or 10 µM paclobutrazol (Pac) for 24 h, respectively. To ensure the hormone/inhibitor application on leaves, the over ground part of each plant was sprayed with corresponding solution after moving, and the excess liquid was blotted by cotton balls. The *C. bungeana* cells and leaves were collected at different time spots according to the experimental design in each treatment, while the leaves of different *Arabidopsis* lines were collected from 5 weeks old plants under normal growth conditions. All the collected samples were stored at − 80 °C until use.

### RNA isolation and cDNA synthesis

Total RNAs of *C. bungeana* were isolated from suspension-cultured cells or the different tissues of regenerated plants (0.1 g each) according to the standard procedure of Plant RNA Kit (Omega, USA). After being treated with RQ1RNase-free DNase (Promega, USA), the purity and integrity of total RNAs were assessed by UV spectrophotometry and agarose gel electrophoresis. Then, the qualified total RNAs were employed in reverse transcription reaction by using PrimeScript™ Reverse Transcriptase (Takara, Japan) and Oligo(dT)_15_ primer (Takara, Japan) following the manufacturer’s instruction. Reverse-transcribed cDNA samples were stored at -20 °C until further use.

### Cloning and bioinformatics analysis of *CbFAD7* and *CbFAD8*

A 732-bp fragment of *CbFAD7* and a 982-bp fragment of *CbFAD8* were cloned from *C. bungeana* by using degenerate primers (P1 and P2 for *CbFAD7*; P3 and P4 for *CbFAD8*; Supplementary Table S1) designed basing on the conserved domain database from tobacco, *Brassica napus* and *Arabidopsis*. Amplification of 5’ and 3’ ends of *CbFAD7* and *CbFAD8* were accomplished using specific primers (P5-P8 for *CbFAD7*; P9-P12 for *CbFAD8*; Supplementary Table S1) and SMARTer™ RACE cDNA Amplification Kit (Clontech, Japan). Full-length cDNA of *CbFAD7* and *CbFAD8* were gotten by using specific primers (P13 and P14 for *CbFAD7*; P15 and P16 for *CbFAD8*; Supplementary Table S1). PCR products were cloned into the pMD-18T vector (Takara, Japan) and sequenced by GENEWIZ Inc. (Suzhou, China). Then these sequences were analyzed by DNAman 5.2.9, MEGA 6.06 and ClustalX 1.83 software. The prediction of transmembrane domain and transit peptide were analyzed by the online server program TMHMM (http://www.cbs.dtu.dk/services/TMHMM/) and TargetP (http://www.cbs.dtu.dk/services/TargetP/), respectively.

### Complementation of *CbFAD7* and *CbFAD8* in *Arabidopsis* mutant

The coding region of *CbFAD7* amplified using specific primers (P17and P18; Supplementary Table S1), were cloned within the XbaI-SacI site of the binary vector pBI121 to replace the GUS gene and construct the recombinant plasmid, pBI121-*CbFAD7*, under the control of CaMV 35S promoter. In the same way, the recombinant plasmid pBI121-*CbFAD8* was constructed with the coding region of *CbFAD8* amplified using specific primers (P19 and P20; Supplementary Table S1). Then, the two recombinant plasmids and the *Arabidopsis* seeds of *fad7fad8* mutant were sent to Shanghai Weidi Biotechnology Co., Ltd (Shanghai, China) to get the complemented mutants, F7 and F8. The transgenic *Arabidopsis* plants were generated through the floral dip by *Agrobacterium*-mediated transformation. Positive transgenic lines of F7 and F8 exhibiting 3:1 segregation ratio were identified by PCR using specific primers (P17 and P18 for *CbFAD7*; P19 and P20 for *CbFAD8*; Supplementary Table S1 and Fig. S1). The homozygous lines were obtained by backcrossing or self-pollination, and the expression level of *CbFAD7* and *CbFAD8* were verified by qRT-PCR using specific primers (P23 and P24 for *CbFAD7*; P25 and P26 for *CbFAD8*; Supplementary Table S1 and Fig. S1), respectively. IPT2 (AT2G27760) was taken as the house keeping gene^70^ using primers P29 and P30 (Supplementary Table S1). Three independent homozygous T3 transgenic lines of F7 and F8 showing higher expression levels were used in the experiments, respectively.

### Quantitative real-time PCR

According to genomic data analysis (data not shown), each *FAD3*, *FAD7* and *FAD8* gene has one single copy in the *Chorispora* genome. The SYBR Green I (Takara, Japan) assay and the Real-Time PCR System (Mx3000P, Agilent Stratagene, USA) were used for detecting the expression of *CbFAD3*, *CbFAD7* and *CbFAD8* in *C. bungeana*. The housekeeping gene, *CbACT* (AY825362), was used as a control for the stable expression^32–34,71^. The amplification specificity of the primers (P21 and P22 for *CbFAD3*; P23 and P24 for *CbFAD7*; P25 and P26 for *CbFAD8*; P27 and P28 for *CbACT*; Supplementary Table S1) were checked by gel electrophoresis before real-time PCR. The amplification condition was as follow: 95 °C for 30 s, and 40 cycles of 95 °C for 5 s and 58 °C for 34 s. This was followed by 15 s at 95 °C, 60 s at 60 °C and 15 s at 95 °C (determination of melting curve). PCR data were obtained from three independent biological samples for each experiment. The relative gene expression (F) was normalized against the housekeeping gene according to the formula: ${\text{F}} = \frac{{\left( {{\text{E}}_{{{\text{tgt }}}} } \right)^{{\Delta {\text{Ct }}_{{{\text{tgt }}}} (ctrl - spl)}} }}{{\left( {{\text{E}}_{{{\text{hk }}}} } \right)^{{\Delta {\text{Ct }}_{{{\text{hk }}}} (ctrl - spl)}} }}$, which was regarded as a high accuracy and reproducibility mathematical model^72^.

### Extraction and analysis of total fatty acids

*C. bungeana* cell suspensions and leaves as well as *Arabidopsis* leaves (2 g each) were grinded with liquid N2, respectively. The lipids and the total fatty acids of each sample were prepared and analyzed as our previous reports^3,17^. The fatty acid methyl esters of each sample were analyzed by GC–MS (6890N-5975C, Agilent, USA) fitted with a capillary column (Agilent DB-FFAP, 30 m × 0.25 mm × 0.5 µm) according to our previous procedure^70^. Fatty acid data were obtained from five independent biological samples for each experiment.

### Extraction and analysis of phytohormones

*C. bungeana* suspension-cultured cells and leaves (0.5 g each) were sent to Genepioneer Biotechnologies (Nanjing, China) to detect the concentration of JA, BRs, ABA and GA3, respectively. Phytohormones were extracted through grinding and organic solvent extraction, and then analyzed using Plant JA ELISA Kit (Sinobestbio, China), Plant BRs ELISA Kit (Sinobestbio, China), Plant ABA ELISA Kit (Sinobestbio, China) and Plant GA3 ELISA Kit (Sinobestbio, China), respectively. During the double-antibody sandwich ELISA, the absorbance (OD value) was detected by microplate reader (Infinite F50, Tecan, SWIT), and the concentration was calculated through standard curve method. Phytohormone data were from five biological replicates for each experiment.

### Statistical analysis

Statistical analysis was performed using the method of one-way ANOVA, followed by Duncan’s multiple range test at the *P* < 0.05 or *P* < 0.01 levels.

### Ethical approval

All procedures of this study, including the experimental research and field studies on *C. bungeana* and *Arabidopsis* as well as the collection of plant material, were conducted in accordance with the relevant institutional, national, and international guidelines and legislation. The wild *C. bungeana* plants were collected from an ice free cirque besides the Glacier No. 1 in Tianshan mountains (Xinjiang province, China), which is not a restricted area for researchers.

## Supplementary Information

Supplementary Information.

## Data Availability

The DNA and protein sequences generated during and/or analysed during the current study are available in the NCBI Genbank repository (https://www.ncbi.nlm.nih.gov/nuccore/orhttps://www.ncbi.nlm.nih.gov/protein/; *CbFAD7*: KY069282; *CbFAD8*: KY069283; AtFAD3: NP180559; BjFAD3: ADJ58019; BnFAD3: NP001302640; BoFAD3: AGH20189; CbFAD3: KM591203; CbFAD7: KY069282; CbFAD8: KY069283; DsFAD3: ABK91879; GmFAD3: NP001237507; LeFAD3: ABX24525; LuFAD3: AFJ53089; NtFAD3: P48626; SaFAD3: AHA05997; TaFAD3: BAA28358; AtFAD7: P46310; BnFAD7: ACS26170; DsFAD7: ABS86961; NtFAD7 D79979; SlFAD7 NP001234592; OsFAD7: BAE79783; AtFAD8: P48622; BnFAD8: NP001302644; BrFAD8: AAW78909; DsFAD8: ABK91881; GmFAD8-1: NP001238609; OsFAD8: BAE79784). The other datasets generated during and/or analysed during the current study are available from the corresponding author on reasonable request.
