# Supplementary material for: Phytohormones regulate the non-redundant response of ω-3 fatty acid desaturases to low temperatures in Chorispora bungeana
Source: Sci Rep. 2023 Feb 16;13:2799. doi: 10.1038/s41598-023-29910-4 (PMC9935925; doi:10.1038/s41598-023-29910-4)
Supplement: Supplementary file 1 — Supplementary Information. [file 41598_2023_29910_MOESM1_ESM.pdf]

# Phytohormones Regulate the Non-redundant Response of ω-3 Fatty Acid Desaturases to Low Temperatures in *Chorisporа bungeana*

**Yulan Shi[a*], Sizhong Yang[b], Zhixing Zhao[c], Lizhe An[c,d]**

a. Extreme Stress Resistance and Biotechnology Laboratory, Northwest Institute of Eco-Environment and Resources, Chinese Academy of Sciences, Lanzhou 730000, China.

b. State Key Laboratory of Frozen Soils Engineering, Northwest Institute of Eco-Environment and Resources, Chinese Academy of Sciences, Lanzhou 730000, China.

c. School of Life Sciences, Lanzhou University, Lanzhou 730000, China.

d. School of Forestry, Beijing Forestry University, Beijing 100083, China

***Corresponding author:** Dr. Yulan Shi

Northwest Institute of Eco-Environment and Resources

Chinese Academy of Sciences

Lanzhou 730000

CHINA

E-mail: sylbaby15@lzb.ac.cn

**Table S1** The information of primers used in the experiment.

| Primer | annotation | Sequence (5’-3’) | Length of production (bp) |
|---|---|---|---|
| P1 | sence | ATCTACAC**M**ACACCCAGATCCA | 1133 |
| P2 | antisence | GGCTCCCT**R**TAATACTTC | |
| P3 | sence | GAGGA**R**GAC**R**CG**R**A**K**AGATT | 982 |
| P4 | antisence | GTAAGTGAAGTGGCAGAGG**R** | |
| P5 | 5’outer | TTTTCTCAGACATAGGATGCCAGGAT | 902 |
| P6 | 5’innner | GAATCTTTGCGTTTCCTCTTCGTGTT | 502 |
| P7 | 3’outer | TGGTGGGTCATATTCTTCATTCCTCA | 1061 |
| P8 | 3’innner | CGTGGGAAGGAATGGAGTTACCTGAGAG | 650 |
| P9 | 5’outer | GAGTGACTTGTTTCTTCCGAAAGAGA | 1139 |
| P10 | 5’innner | CGACGCAAATGTTTAGGTTTACACTG | 1036 |
| P11 | 3’outer | GCGACGCAAATGTTTAGGTTTACAC | 609 |
| P12 | 3’innner | TCCCTTGTTTGCCTCAACTTTGTCA | 432 |
| P13 | sence | ATTTGTGTAGAAgCACAGAGAAACAGA | 1805 |
| P14 | antisence | GTTTGAGGTTTAATCAACCAATCCAAAA | |
| P15 | sence | TGTGTAGACACCACAGACCACG | 1563 |
| P16 | antisence | AAGACCGAACGATCCAAATTTTAAGAAC | |
| P17 | sence | GC TCT AGA ATG GCG AAC TTG GTC TTA TCA G | 1320 |
| P18 | antisence | C GAG CTC TTA CAC CTT GAT TTC TCC ATA GAG | |
| P19 | sence | GC TCT AGA ATG GCG AGT TGG GTT TTA TCA | 1194 |
| P20 | antisence | C GAG CTC TCA CCA AGA TCC AGA GTT TTT CG | |
| P21 | sence | CCACGAAGGCAGCGAAA | 96 |
| P22 | antisence | TAGCGACCAAACTCTCAACCAA | |
| P23 | sence | GGACCTTTGCCATTACACTTAT | 151 |
| P24 | antisence | CCTGCTTCATTTCAATCTGC | |
| P25 | sence | AATGGCGAGTTGGGTTTT | 128 |
| P26 | antisence | AAGGAGGTGTAATTGGTGGAT | |
| P27 | sence | ATACGCTCTTCCACACGCTATT | 125 |
| P28 | antisence | TCACGATTTCACGCTCTGCT | |
| P29 | sence | GGATACCGAGGAGTGTTGTGC | 120 |
| P30 | antisence | GGTCAACCACTGTCGGCATA | |

**Table S2** The main fatty acid contents of total lipids from *C. bungeana* suspension-cultured cells under different low-temperature treatments.

| **Temperature (℃)** | **Treated time (h)** | **C16:0** | **C18:0** | **C18:1** | **C18:2** | **C18:3** |
|---|---|---|---|---|---|---|
| **25** | **0** | 30.41±1.12 | 10.73±0.05 | 15.25±0.51 | 23.13±0.84 | 20.48±0.95 |
| | **6** | 29.93±0.43 | 9.69±0.26 | 15.75±0.43 | 24.42±0.55 | 20.21±0.88 |
| | **12** | 30.26±0.69 | 9.25±0.09 | 15.66±0.62 | 22.35±0.98 | 22.51±0.63 |
| | **24** | 31.15±0.38 | 9.98±0.31 | 15.03±0.47 | 24.53±1.26 | 19.29±1.35 |
| **4** | **0** | 30.34±1.30 | 10.06±0.40 | 15.18±0.45 | 22.78±0.65 | 21.65±1.17 |
| | **6** | 29.69±0.57 | 8.58±0.01 | 11.23±0.41 | 15.69±1.09 | 34.83±1.25 |
| | **12** | 28.03±0.46 | 3.95±0.06 | 6.62±0.23 | 11.21±0.92 | 50.22±1.76 |
| | **24** | 29.86±0.49 | 5.18±0.23 | 10.92±0.09 | 23.95±1.00 | 30.11±0.96 |
| **0** | **0** | 30.07±1.26 | 10.13±0.09 | 15.11±0.18 | 22.67±1.20 | 22.05±1.47 |
| | **6** | 29.83±0.63 | 5.92±0.01 | 10.33±0.30 | 15.02±0.51 | 38.92±0.95 |
| | **12** | 28.69±0.32 | 2.47±0.02 | 4.62±0.26 | 9.23±0.44 | 55.01±1.18 |
| | **24** | 30.05±1.21 | 6.11±0.48 | 9.25±0.39 | 10.06±0.56 | 44.53±0.55 |
| **-4** | **0** | 30.18±0.66 | 10.20±0.22 | 15.28±0.50 | 23.01±1.11 | 21.36±0.65 |
| | **6** | 29.87±0.39 | 6.79±0.39 | 10.76±0.10 | 14.35±1.02 | 38.23±0.97 |
| | **12** | 29.76±0.45 | 6.34±0.10 | 6.52±0.12 | 11.17±0.87 | 46.24±1.56 |
| | **24** | 29.60±0.52 | 6.02±0.11 | 6.35±0.22 | 15.41±0.99 | 42.63±1.15 |

**Table S3** The main fatty acid contents of total lipids from *C. bungeana* leaves under different low-temperature treatments.

| Temperature (℃) | Treated time (h) | C16:0 | C16:1 | C16:2 | C16:3 | C18:0 | C18:1 | C18:2 | C18:3 |
|---|---|---|---|---|---|---|---|---|---|
| **25** | **0** | 18.34±0.58 | 0.65±0.26 | 0.63±0.25 | 2.89±0.13 | 1.71±0.33 | 6.11±0.75 | 21.88±0.89 | 47.81±0.31 |
| | **6** | 18.11±0.44 | 0.44±0.10 | 0.40±0.18 | 2.51±0.26 | 1.58±0.45 | 7.77±0.87 | 24.11±1.49 | 45.10±1.36 |
| | **12** | 18.30±0.45 | 0.47±0.19 | 0.43±0.16 | 2.94±0.18 | 1.26±0.42 | 7.33±0.85 | 23.54±0.87 | 45.73±0.63 |
| | **24** | 18.19±0.39 | 0.54±0.08 | 0.51±0.11 | 2.72±0.05 | 1.47±0.15 | 6.86±0.44 | 23.19±0.38 | 46.42±0.69 |
| **4** | **0** | 17.62±0.49 | 0.57±0.13 | 0.52±0.06 | 2.88±0.14 | 1.41±0.30 | 6.76±0.61 | 23.07±0.21 | 47.18±0.20 |
| | **6** | 18.39±0.61 | 0.57±0.14 | 0.37±0.12 | 3.44±0.15 | 1.50±0.14 | 4.70±0.45 | 18.07±1.75 | 52.93±1.10 |
| | **12** | 17.96±0.25 | 0.44±0.16 | 0.34±0.05 | 5.15±0.08 | 0.90±0.22 | 2.98±0.35 | 11.58±0.16 | 60.68±0.03 |
| | **24** | 19.22±0.54 | 0.84±0.27 | 0.54±0.24 | 2.28±0.24 | 1.20±0.09 | 4.87±0.49 | 19.69±0.33 | 51.36±0.81 |
| **0** | **0** | 18.72±0.65 | 0.61±0.15 | 0.60±0.19 | 2.68±0.10 | 1.69±0.51 | 6.38±0.72 | 22.26±0.76 | 47.07±0.26 |
| | **6** | 17.99±0.44 | 0.53±0.24 | 0.36±0.18 | 3.37±0.23 | 1.06±0.45 | 3.91±0.35 | 13.90±0.99 | 58.91±0.58 |
| | **12** | 17.87±0.18 | 0.40±0.23 | 0.27±0.21 | 4.25±0.30 | 0.87±0.44 | 3.79±0.65 | 11.89±1.34 | 60.70±1.12 |
| | **24** | 18.01±0.24 | 0.45±0.09 | 0.34±0.22 | 3.94±0.28 | 1.01±0.21 | 3.87±0.46 | 12.00±1.58 | 60.39±1.21 |
| **-4** | **0** | 17.66±0.53 | 0.54±0.11 | 0.52±0.07 | 2.78±0.13 | 1.41±0.45 | 6.77±0.30 | 23.11±0.61 | 47.13±0.13 |
| | **6** | 17.49±0.65 | 0.49±0.21 | 0.45±0.23 | 3.74±0.27 | 1.25±0.28 | 5.12±0.39 | 17.81±0.82 | 53.68±1.61 |
| | **12** | 18.11±0.55 | 0.35±0.06 | 0.33±0.13 | 4.08±0.20 | 1.37±0.19 | 3.94±0.25 | 13.23±0.54 | 58.61±0.36 |
| | **24** | 17.77±0.31 | 0.33±0.13 | 0.27±0.12 | 5.03±0.38 | 1.43±0.25 | 4.01±0.68 | 14.29±1.37 | 56.89±1.42 |

**Table S4** The main fatty acid contents of total lipids from *C. bungeana* suspension-cultured cells under cold and phytohormone inhibitor treatments.

| **Treatment (12h)** | **C16:0** | **C18:0** | **C18:1** | **C18:2** | **C18:3** |
|---|---|---|---|---|---|
| **Control** | 30.26±0.69 | 9.25±0.09 | 15.66±0.62 | 22.35±0.98 | 22.51±0.63 |
| **0℃** | 28.69±0.32 | 2.47±0.02 | 4.62±0.26 | 9.23±0.44 | 55.01±1.18 |
| **0℃+DIECA** | 29.95±0.75 | 8.81±0.11 | 11.34±0.45 | 15.60±0.89 | 34.32±0.94 |
| **0℃+Pcz** | 29.68±0.94 | 9.15±0.32 | 12.96±0.19 | 15.79±1.02 | 32.44±0.43 |
| **0℃+DIECA+Pcz** | 30.14±1.10 | 9.67±0.15 | 15.52±0.51 | 20.84±0.95 | 23.85±0.74 |

**Table S5** The main fatty acid contents of total lipids from *C. bungeana* leaves under cold and phytohormone inhibitor treatments.

| Treatment (12h) | C16:0 | C16:1 | C16:2 | C16:3 | C18:0 | C18:1 | C18:2 | C18:3 |
|---|---|---|---|---|---|---|---|---|
| **Control** | 18.30±0.45 | 0.47±0.19 | 0.43±0.16 | 2.94±0.18 | 1.26±0.42 | 7.33±0.85 | 23.54±0.87 | 45.73±0.63 |
| **0℃** | 17.87±0.18 | 0.40±0.23 | 0.27±0.21 | 4.25±0.30 | 0.87±0.44 | 3.79±0.65 | 11.89±1.34 | 60.70±1.12 |
| **0℃+DIECA+Pcz** | 18.38±0.69 | 0.41±0.15 | 0.35±0.10 | 3.69±0.10 | 1.49±0.17 | 5.54±0.48 | 18.01±1.15 | 52.14±0.64 |
| **0℃+DIECA+Pcz+Pac** | 18.34±0.37 | 0.42±0.13 | 0.39±0.13 | 3.34±0.05 | 1.30±0.26 | 7.01±0.66 | 20.97±0.91 | 48.23±0.42 |
| **0℃+DIECA+Pcz+Pac+Flu** | 18.15±0.48 | 0.44±0.11 | 0.41±0.19 | 2.98±0.12 | 1.46±0.47 | 7.47±0.81 | 23.88±1.40 | 45.21±0.39 |

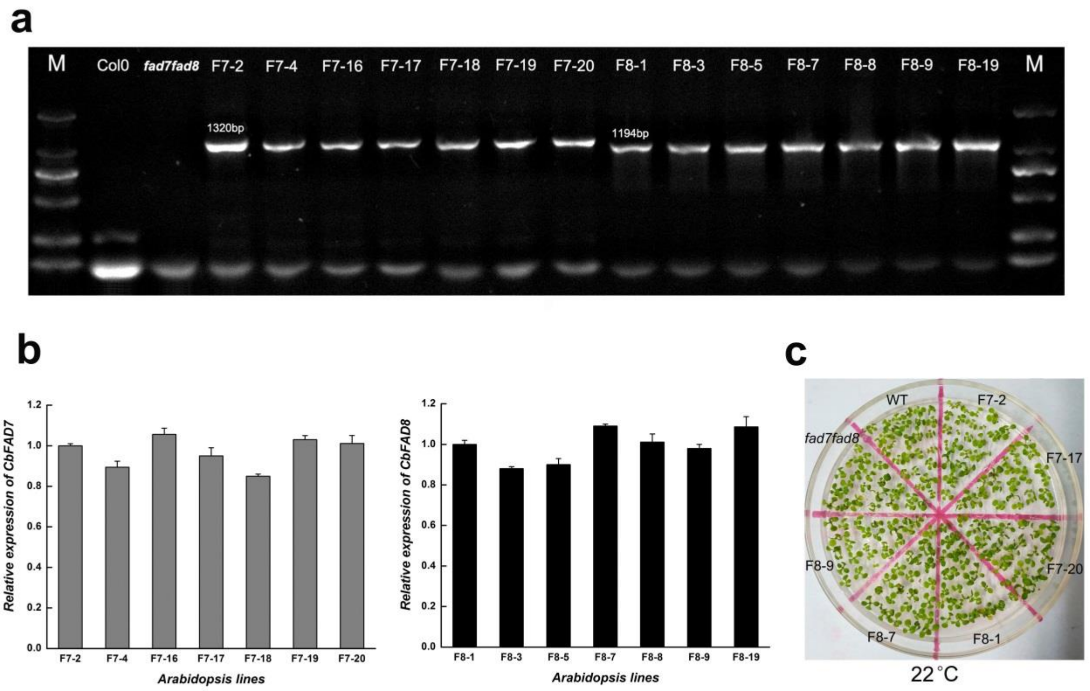


**Fig. S1**

Molecular and germination analyses of *Arabidopsis* lines. (a) PCR analysis of the coding region of *CbFAD7* (1320bp) and *CbFAD8* (1194bp) in WT plants, *fad7fad8* mutants and complemented mutants. (b) Real-time PCR analysis of the expression of *CbFAD7* and *CbFAD8* in complemented mutants. (c) Germination of *Arabidopsis* lines under normal conditions. The photograph was taken 25 d after vernalization. The *CbFAD7*-complemented and the *CbFAD8*-complemented mutants were represented as F7 and F8, respectively. Each value represents the mean ± SE of three replicates.

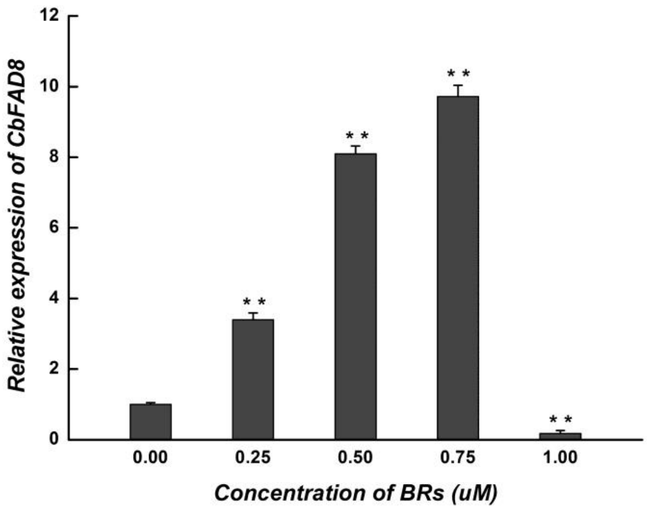


**Fig. S2**

Effect of the concentration of exogenous BRs on the expression of *CbFAD8* in *C. bungeana* cell suspensions. The expressions of *CbFAD8* were detected at being treated for 3 h. Data were presented as relative expression ratios being compared with the expression level of corresponding gene without BRs treatment, which were set at a value of 1. Each value represents the mean ± SE of three replicates.

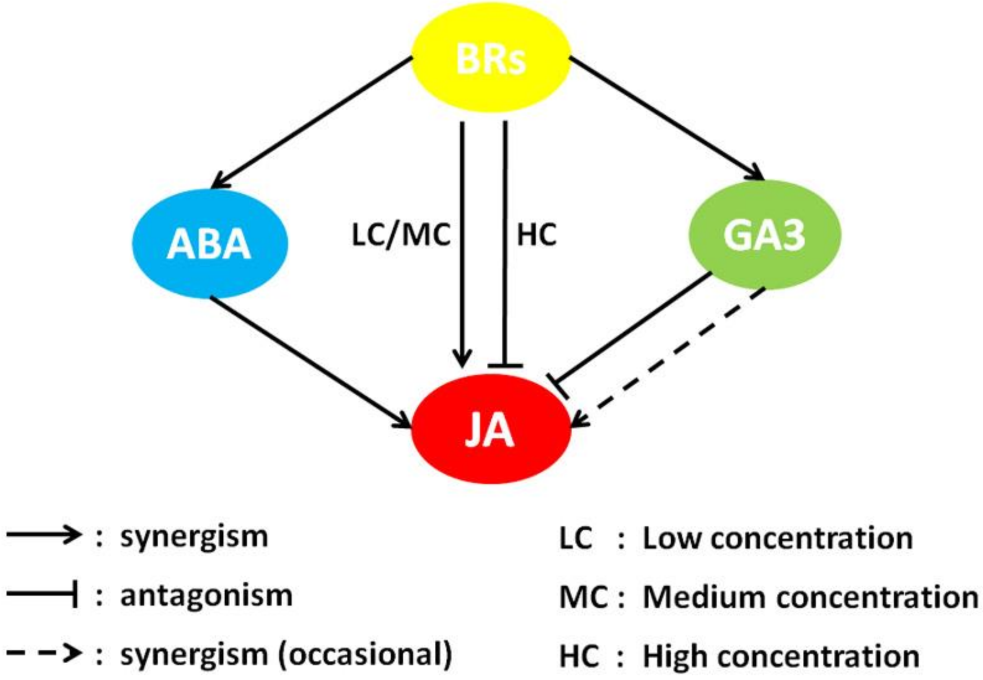


**Fig. S3**

Schematic diagram of the interaction between JA, BRs, ABA and GA3.

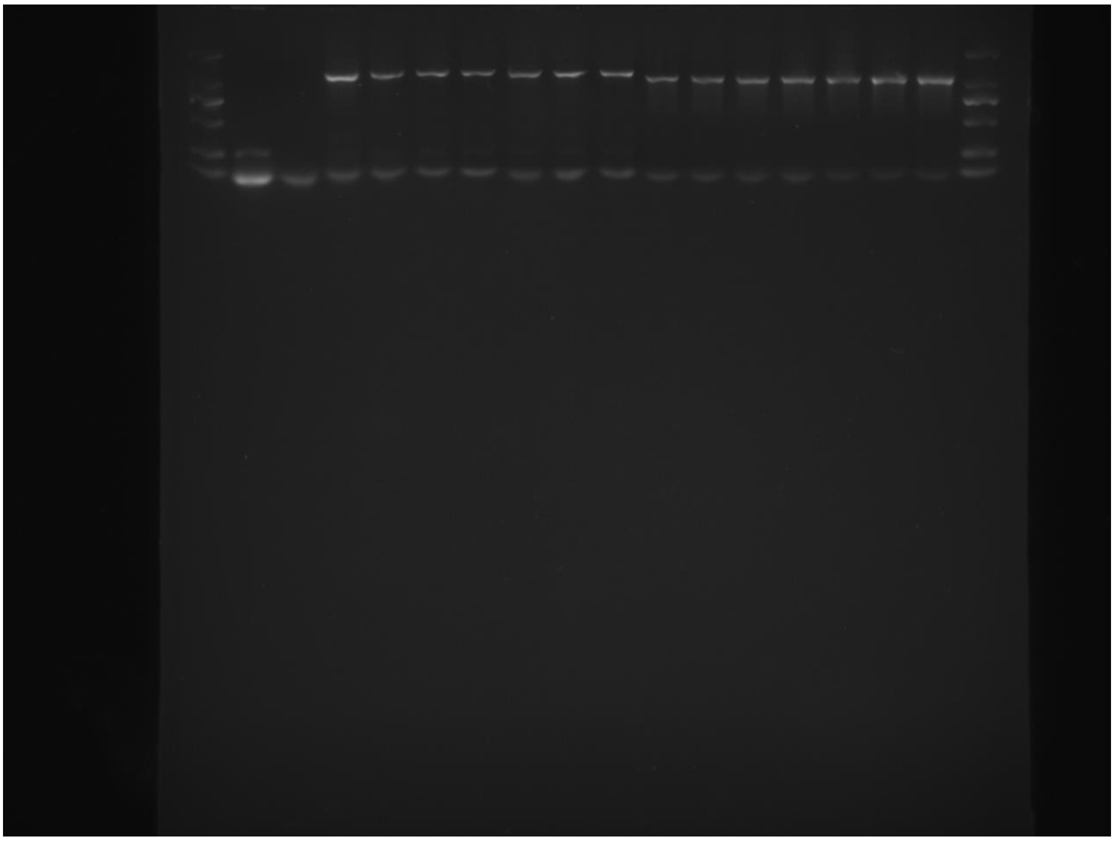

**Fig. S4**

The original gels image used in Fig. S1a. Before it been used in Fig. S1, fine adjustments have been done through Adobe Photoshop 9.0. First, the contrast of the original photo has been enhanced to make it clearer. Second, the irregular part (about 2/5) of the left DNA marker has been cut, and the regular part has been brought out mirror symmetry to make it more neat and beautiful. At last, the picture has been labeled and cut into suitable size.
